# National surgical, obstetrics, and anaesthesia plans in Sub-Saharan Africa: a comparative economic content analysis

**DOI:** 10.1186/s12913-026-14981-6

**Published:** 2026-06-29

**Authors:** Martilord Ifeanyichi, Phyllis Tenkorang, Pedra Rabiee, Alshaheed Nasraldin Abdelhabeeb, Meskerem Kebede, Maeve Bognini, Ryan Ellis, Rachel Hargest, Rocco Friebel

**Affiliations:** 1 Global Surgery Policy Unit, LSE Health, London School of Economics and Political Science, Cowdray House, Houghton Street, London, WC2A 2AE UK; 2 Global Anaesthesia, Surgery and Obstetric Collaboration, Newcastle, UK; 3 African Centre for Health Economics and Policy Research, Enugu, Nigeria; 4 Department of Anaesthesia and Intensive Care, Korle Bu Teaching Hospital, Accra, Ghana; 5 Department of Obstetrics and Gynaecology, Addenbrooke’s Hospital, Cambridge University Hospitals NHS Foundation Trust, Cambridge, UK; 6 Department of Anaesthesiology, The Royal London Hospital, London, UK; 7 School of Medicine, University Hospital of Wales, Cardiff, CF14 4XN UK; 8 Royal College of Surgeons of England, London, UK; 9 Department of Health Policy, London School of Economics and Political Science, London, WC2A 2AE UK

**Keywords:** Global surgery, Health economics, Health policy, Sub-Saharan Africa, NSOAP, Content analysis, Financing

## Abstract

**Introduction:**

The introduction of National Surgical, Obstetrics, and Anaesthesia Plans (NSOAPs) in Sub-Saharan Africa (SSA) aims to address the region’s significant gaps in access to surgical care. The financial implications of these plans, which are crucial for implementation and scaling, remain underexplored. This study presents a comparative analysis of the economic content of NSOAPs across ten SSA countries, highlighting financial strategies, costing, and proposed implementation frameworks.

**Methods:**

A mixed-methods policy content analysis was conducted. Data were collected from published documents, followed by a qualitative thematic analysis of economic components. Quantitative analysis focused on the comparison of total and per capita costs, adjusted for purchasing power parity (i.e., in International dollars [I$]), and aligned against socio-economic indicators.

**Results:**

There was significant variability in the economic content covered across the ten NSOAPs. Overall, most plans emphasised resource mobilisation and to a lesser extent financial protection, while purchasing was the most neglected. Nigeria and Tanzania provided the most comprehensive financing strategies, including innovative resource mobilisation, risk protection, and purchasing (at least implicitly); other countries like Rwanda and Madagascar lacked detailed financial protection and purchasing mechanisms. Total lifespan costs ranged from I$221 million (Senegal) to I$50.5 billion (Nigeria), with per capita costs varying widely. Annual costs as a percentage of Gross Domestic Product were modest, but in some countries, like Nigeria, implementation would require nearly 10% of the annual national budget. Involvement of (health) economics specialists and ministry of finance stakeholders was notably low, generally.

**Conclusion:**

NSOAPs represent an important policy tool for improving surgical care in SSA. However, inadequate economic planning and financing strategies present significant barriers to their implementation. Future plans should be developed through multi-disciplinary expertise and inter-sectoral engagement with a focus extending beyond resource mobilisation to encompass financial protection and purchasing to ensure sustainable investments in surgical care. Unbundling the plans into smaller, contextually prioritised packages may enhance their perceived financial feasibility and, consequently, their appeal to governments and funders.

**Supplementary Information:**

The online version contains supplementary material available at https://doi.org/10.1186/s12913-026-14981-6.

## Introduction

Within the last decade, many countries across sub-Saharan Africa (SSA) have witnessed an increasing momentum around surgical care. This is due to triple events recorded in 2015, including the passing of World Health Assembly resolution 68.15 to officially adopt surgery as an indispensable component of universal health coverage (UHC) [1]; the publication of Volume 1 of the third edition of the World Bank Disease Control Priorities (DCP3) focusing entirely on essential surgery [2]; and the publication of the Lancet Commission on Global Surgery (LCGS) report highlighting large inequities in access to surgery with corresponding health and economic implications [3]. The LCGS provided a blueprint, in the form of National Surgical, Obstetrics, and Anaesthesia Plans (NSOAPs), otherwise simply called surgical plans, for harnessing resources and stakeholders towards the scale-up of surgical care. Plans commonly provide a context-specific situational analysis of surgical care and offer a roadmap for scale-up of surgical services, ideally embedded with the broader health system context. Mirroring the health systems’ building blocks, NSOAPs are structured around five main domains: infrastructure, service delivery, human resources, information systems, governance/leadership, and financing [3].

In the period from the publication of the LCGS report in 2015 until December 31, 2024 (the cut-off point for this study), a total of nine sub-Saharan African countries (i.e., Zambia, Tanzania, Nigeria, Madagascar, Ethiopia, Rwanda, Zimbabwe, Namibia, and Ghana) developed and launched at least the first iteration of an NSOAP. Ethiopia launched its second national surgical plan in 2020, following the expiration of the first plan (2016–2020), while Zambia and Nigeria are currently revising their first plans as a preamble to the launch of the second plans. Furthermore, between January 2025 and now (June 2026), Kenya and Sierra Leone have launched national surgical plans. Several other countries are developing NSOAPs, or have expressed intentions to do so. Meanwhile, Senegal had developed a surgical plan in 2013, (i.e., two years prior to the publication of the LCGS report), suggesting that national surgical planning was not necessarily a novel concept in Africa. The United Nations Institute for Training and Research and other collaborators developed a manual for national surgical planning in LMICs in 2020 [4]. However, while there have been significant expectations from the surgical plans to mobilise investments and channel resources into the strengthening of surgical care [5], there are indications that efforts to implement the NSOAPs have encountered a series of multi-faceted challenges [6], with funding emerging as a prominent constraint [7].

There is paucity of research with focus on NSOAPs. Existing publications of mostly non-empirical evidence emphasise the importance of NSOAPs as pathways for the achievement of universal access to surgery at country levels [8–10], outlining the best practices in the development of NSOAPs [4, 6], and proffering pathways for sustainable financing of the plans [11, 12]. Others reports reflect on the process of developing NSOAPs [8, 13], with a study by Abebe et al., exploring the current landscape of NSOAPs in SSA as well as early lessons from their implementation [14]. There have been only few attempts at analysing the NSOAP content critically [15, 16], and the economic content that includes the costing and proposed surgical financing strategies have been particularly neglected.

Surgical systems financing is concerned with the mobilisation, accumulation, and allocation of money to cover the surgical needs of the people, individually and collectively, in the health system [17]. Experts delineate three core sub-functions: resource mobilisation, resource pooling/prepayment, and purchasing. Resource mobilisation refers to various ways of raising revenues for health care, including through government budgetary allocations (from taxes for instance), health insurance premiums, out-of-pocket (OOP) payments and donor funding. The pooling function accumulates pre-paid funds on behalf of the population to avert financial challenges at the point of access to care. This provides protection against catastrophic and impoverishing expenditures, for example, through health insurance schemes. Purchasing is concerned with various approaches to payment of providers for services and ideally seeks to optimise efficiency through value-based purchasing strategies [17].

Policy document analysis is a research tool that facilitates the examination of a policy background to understand the situational imperatives including values surrounding its creation; an unpacking of the construction and elements of the policy; and an evaluation of the policy modus operandi, its ability for implementation, and the potential to achieve the intended objectives [4]. Especially as every domain of the NSOAP depends on funding to be mobilised, an economic content analysis may offer valuable insights into the reasons why NSOAPs have been unable to present as a step change for strengthening surgical care across SSA to address the high surgical burden [18]. This study provides a comparative analysis of the economic content of ten NSOAPs to identify possible barriers and potential opportunities for their successful implementation in the future.

## Methods

A mixed method (i.e., quantitative and qualitative) policy content analysis was employed. We adopted a broad approach based on the understanding that “all aspects of how a policy comes into being and how it is translated into practice are important” [4]. All NSOAPs that had been launched in SSA until December 31, 2024, were retrieved via web searches, without any time or language restrictions. Plans that were not published in English language were translated using the translation tool Google Translate.

### Qualitative analysis

A qualitative document analysis was conducted to synthesise all economic (i.e., financing and costing) elements specified in the plans, using a combination of inductive and deductive approaches. In the inductive analysis, two researchers working in parallel read closely all the NSOAPs to identify and code words and phrases with economic connotations. Emerging themes and patterns were identified and related themes were grouped into broader themes. After the coding of each NSOAP, a meeting was held between the coders and a senior researcher to review and evaluate the agreement in the codes and harmonise any discrepancies. To ensure that all areas in the NSOAP related to financing and costing were identified, documents were text-field searched (using “finance/ing”, “cost/ing”, “health/ economi*”, and ‘’Ministry of Finance’’). A similar analytical approach was previously applied in the study of the inclusion of children care in NSOAPs in SSA [6].

During the deductive stage, the inductively identified themes were mapped into a pre-determined Cardno framework [19]. In addition, other non-economics data related to different domains of the framework were extracted from the NSOAPs. This framework contains five domains: (i) document production and location; (ii) authorship and audience; (iii) policy context; (iv) policy text; and (v) policy consequences [4]. Details of the framework including illustrative questions probed in each domain are provided in Supplementary file 1.

For *document production and location*, it was assumed that the capital cities of the study countries were the respective places of production of the documents, while the sources of the NSOAPs (i.e., online versus personal contacts) were documented. Regarding *authorship and audience*, we conducted a critical analysis of all the contributors (i.e., individuals, organisations, and entities) to the plan development (where available) to assess the diversity, and more specifically, the involvement of non-surgeons or non-clinicians such as (health) economists and public health experts. For the participants, we used the designations/position/role where available in the document. In addition, we searched the names on Research Gate, LinkedIn, Google Scholar, and the general Google search engine to determine individuals’ main professional backgrounds. Contributors were classified based on their primary work role, for instance, medical doctors working in public health were classified as public health experts. Under *policy context*, we explored the baseline situation that informed the development of the plan, and while the financing domain was the primary focus, the indicators related to other surgical systems domains were also extracted for a deeper appreciation of the background. With *policy text and policy consequences*, we articulated the key elements of the plans in the financing domain as informed by the inductive analysis.

The qualitative data related to financing were summarised using a narrative review while maintaining the same over-arching framework. Sub-themes relating to the costing of the plans were isolated and reported with the quantitative analysis results.

### Quantitative analysis

Our approach builds on the analysis of Jumbam et al. (2019) [16]. For each country, the total and annual costs were compared against a set of socioeconomic indicators, including population, gross domestic product (GDP), GDP per capita, total annual health expenditure, annual budgetary allocation to health, and domestic general government expenditure on health. To facilitate this comparison across years and countries, all costs were first converted to 2022 equivalents using the World Bank GDP deflator [11], and then expressed as International dollars (I$) using International Monetary Fund purchasing power parity (PPP) converters [12].

## Results

**Fig. 1:**
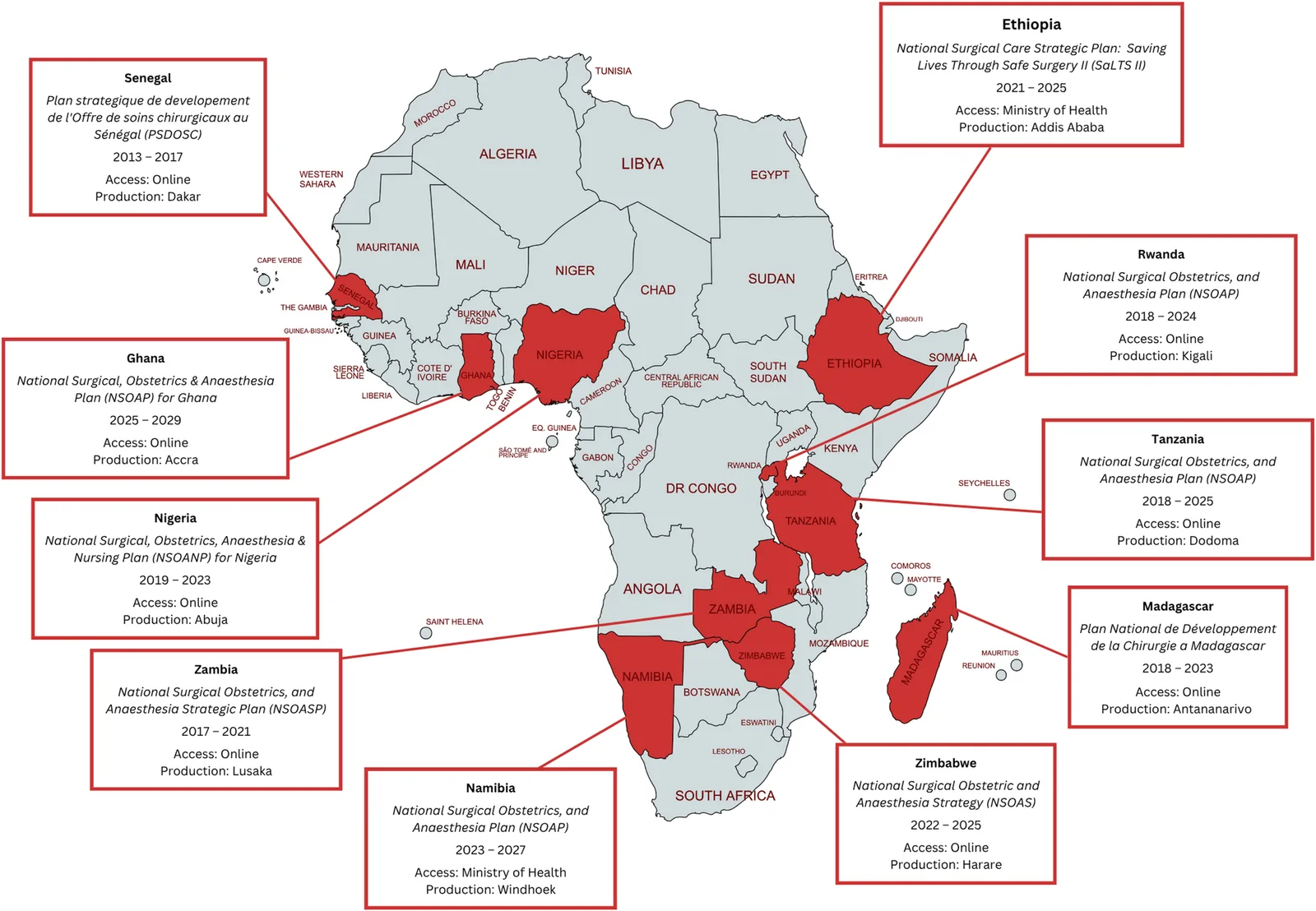
Geographic overview of National Surgical Obstetrics and Anaesthesia Plans across Sub-Saharan Africa, as of December 31, 2024

A total of ten surgical plans were analysed. A geographic overview of all the NSOAPs is presented in Fig. 1.

All the plans (except Zimbabwe and Madagascar) provided the list of individuals (and organisations) that contributed to the NSOAP development, with various degrees of detail about their professional backgrounds. Overall, most of the participants had primary qualifications as medical doctors (most of whom were practicing surgery, obstetrics and anaesthesia specialists). In Nigeria for instance, seven out of eight of the core development committee membership and 11 of 13 writing committee membership had medical backgrounds, and about 90% were surgeons. The other medically qualified participants were experienced or trained in public health, health systems strengthening, health administration or hospital management. Of note, only three countries included a formally trained health economist (Senegal, Tanzania and Rwanda) or economist (Tanzania) in the core team.

In all the countries, the development of the plan was superintended by the respective Ministries of Health, particularly the directorates of hospital, curative, clinical, or medical services. Evidence of intra-ministerial inter-departmental collaboration was found in most of the plans. For instance, this included an involvement of the department of human resources; Ethiopia included an architect from the public health infrastructure directorate. However, intersectoral or inter-ministerial engagement was low. Only Nigeria explicitly reported the involvement of the Ministry of Finance. Our analysis identified diverse partners that supported the development of NSOAPs, ranging from local professional associations to international organisations. The most notable of the international collaborators is the Harvard Medical School Programme for Global Surgery and Social Change (PGSSC) which was formally acknowledged in six (60%) out of the ten plans studied.

### Policy context

Details of the baseline situation across all surgical systems indicators are provided in Table 1. Except for Madagascar and Senegal, all plans described the health insurance coverage, proportion of out-of-pocket expenditure, risk of catastrophic expenditure, and risk of impoverishing expenditure, with large variation across countries. For example, Nigeria reported 73% out-of-pocket expenditure with only 12% of the public expenditure attributed to surgery and less than 3% of Nigerians covered by health insurance. Rwanda reported an 11% risk of catastrophic expenditure due to surgery among its population with a total of 91% national health insurance coverage (85% from Community-Based Health Insurance [CBHI] and 6% from private insurance). Tanzania reported an 85% risk of impoverishing expenditure and 65% catastrophic expenditure, Zimbabwe reported only 9% catastrophic expenditure with an estimated 6.2% of the 2022 budget attributable to surgery. It is noteworthy that none of the plans provided any information on the baseline situation around the purchasing aspects of the financing of surgical care.

### Policy text and consequences

Overall, all NSOAPs reference broader frameworks such as national health policies or national health sector development strategic plans, with clearly outlined over-arching “goals”, “objectives” or “strategies” and sub-sets of “actions”, “activities” or “interventions” to achieve these goals. (see Supplementary file 2).

#### Senegal

Generally, the Senegal plan, being the first of its kind is remarkably different in structure and scope from the post-2015 plans. The planning does not follow the typical surgical systems domains for instance, but it is instead structured around three core objectives: (i) enhance availability and accessibility of care (ii) improve the organisation of surgical care (iii) strengthen partnerships for surgical care delivery. Regarding financing, the plan identifies private-public partnership (PPP) as a key strategy to mobilise extra resources to scale up the supply of financially accessible surgical services. The plan highlights the number of contracts/agreements shared with private entities and the share of the private partners in the funding of the plan as the progress trackers.

#### Ethiopia

The National Surgical Care Strategic Plan: Saving Lives Through Safe Surgery II (SaLTS II) of Ethiopia, like the Senegal plan, was not structured around the surgical systems framework, but rather on five overarching objectives: (i) access to surgical care; (ii) efficiency; (iii) effectiveness; (iv) people-oriented surgical care; and (v) reduce harm. While the plan provides detailed activities and targets for these objectives, there is a lack of focus on financing functions except for its intentions to “provide and allocate financial support at all levels of the health system” and “promote PPP models to ensure equitable and affordable surgical care (flagship initiative)”. While the plan does not state any explicit financial protection objectives or activities, it identifies National Health Accounts (NHA) as a health financial burden assessment tool that will be deployed in monitoring progress in household protection from financial toxicities due to surgery. Quantitative targets were not set, however.

#### Zambia

The National Surgical, Obstetric, and Anaesthesia Strategic Plan (NSOASP) of Zambia focused strongly on resource mobilisation for surgery, proposing to improve funding levels by, among others, creating dedicated budget lines for surgery at the district levels (level 1) and improving allocation to surgery budget lines at levels 2 and 3. In addition, it sought to establish the true costs of surgical care and to embed a system that would be able to track surgical expenditure across all levels of the healthcare system, with a target of setting up tracking mechanisms at 80% of all the facilities by 2021. However, there was a lack of information on financial risk protection and purchasing functions.

#### Tanzania

The plan aims to extend financial protection to 70% of the population even as it targeted an increase in social health insurance enrolment from 19% in 2015 to 50% by 2020, as well as expanding surgical care coverage in the health insurance scheme. The proposed fund-raising strategies encompassed diversification through the enhancement of the insurance enrolment; the creation of a Trauma Fund financed by motor insurance companies and allocation from road funds; collaboration with private entities; and a planned budgetary allocation increase from 9.1 in 2015 to 10.0% in 2020. Further, the plan proposes the creation of a dedicated district budget line targeting surgical care. Appropriate monitoring metrics such as the risk of catastrophic expenditures, domestic funds committed to surgical, obstetric and anaesthesia (SOA) care at the national and district levels, and amounts of alternative funding sources secured were also stipulated. In the purchasing domain, the plan sought to determine and align actual costs with reimbursements to foster sustainable surgery scale up.

#### Nigeria

The Nigerian National Surgical, Obstetrics, Anaesthesia and Nursing Plan (NSOANP) articulated clear and relatively comprehensive goals in the financing domain, spanning resource mobilisation, risk protection and purchasing. It aimed to increase surgical resources through increased budgetary allocation (up to 10%) and exploration of innovative financing options (including Global System for Mobile Communications [GSM] taxes and sin taxes on alcohol and tobacco), as well as improving financial risk protection by expanding health insurance to up to 40%. In addition, the document aims to optimise benefits from available resources by entrenching value-driven investments and cost-cutting practices. It also outlined several progress trackers, including catastrophic expenditures, percentage budgetary allocation to health and out-of-pocket expenditures.

#### Madagascar

The Malagasy plan emphasised optimising the management of usual sources of resources through various means, including increasing the budgetary allocation to 16% in line with the Abuja Declaration [20] and encouraging hospitals to devise innovative revenue-generating strategies. Furthermore, the document intends to enhance efficiency and accountability in health resource spending through training of hospital managers in budget planning and implementation, and intensification of financial monitoring, auditing, and supervision mechanisms at public hospitals. While the plan did not capture financial protection strategies, it envisaged encouraging the participation of “centres des traitement à domicile” (home treatment centres) in the management of medical care to ease financial challenges, particularly for the poor.

#### Rwanda

The Rwandan NSOAP provides little detail on the objectives, strategies, targets, and monitoring systems. The over-arching financing aspirations expressed in the document were to decrease impoverishing and catastrophic expenditure for patients undergoing surgery. The proposed financing mechanisms entailed augmenting the hospital budget for surgery and ensuring comprehensive coverage of essential surgical procedures within the CBHI. It also committed to intensifying resource mobilisation of resources for NSOAP implementation and instituting tracking and auditing mechanisms for expenditures. To monitor the costs of surgeries, it proposed to establish and periodically review cost indices for surgical procedures across different regions.

#### Zimbabwe

Similar to the Zambian NSOASP, the Zimbabwe National Surgical, Obstetric and Anaesthesia Strategy (NSOAS) was brief in the financing domain, though it provided a clear cardinal goal of improving funding levels and establishing budget lines and systems for resource tracking for SOA services to achieve UHC. It set out to expand the fiscal space for surgery by first establishing the actual costs of surgical services to determine funding requirements, and then enhancing revenues from health insurance while optimising efficiency in the use of available resources. Also, while the plan lacks prepayment strategies, a monitoring and evaluation framework was proposed that uses NHAs to monitor the risk of catastrophic expenditure, which it defined as expenditure of more than 25% of the monthly income on surgical services.

#### Namibia

Although the Namibia plan follows the traditional building blocks, the financing domain was dropped from the framework. The four domains of the plan are: (1) infrastructure and equipment; (2) surgical workforce; (3) service delivery and supplies; and (4) information management. Financing function was marginally captured under the service and supplies domain as the plan seeks to evaluate and respond to patients’ impoverishments due to surgical services and track progress by using the proportion of facilities reporting patient-borne surgical costs.

#### Ghana

The Ghana NSOAP provides little detail on the financing domain, with an overarching objective of providing sustainable financing for surgical care through diversified funding mechanisms and deployment of cost-effective care models. It seeks to achieve this through two key pillars: (i) enhancing affordability of safe, effective and high-quality surgical care and protecting patients against catastrophic and impoverishing health expenditure; (ii) improving resource mobilization and budget allocation for surgery. To facilitate the implementation of the NSOAP, it proposes to develop a resource mobilisation plan and provide subvention for implementation and sustainability of the plan.

### Costs and costing analysis

All NSOAPs provided cost data, except the Zimbabwe plan for which extra costing documents were obtained from an in-country contact with links to the Ministry of Health. Senegal, Zambia, Tanzania, and Rwanda plan provided top-level cost summaries as well as detailed activity-based costs while Ethiopian and Nigerian plans present only top-level figures. The Madagascar document contains only detailed costing data without a summary. In addition, Senegal, Tanzania, and Rwanda provide various degrees of insights into the methodologies and assumptions underlying the cost estimates.

Table 2 provides an overview of the total costs of implementation of the surgical plans in International dollars and placed within local socio-economic contexts. There was large variation in total life span plan costs across countries, ranging from I$221.30 million for a 17.30 million population in Senegal to I$2.20 billion for a 33.10 million population in Ghana. The cost of the Nigerian plans stood out at I$50.50 billion for a 218.50 million population. Accounting for differences in population sizes, the per capita cost of the Nigerian plan was still highest at I$231 per capita, while the cost for all other plans varied from I$2.03 in Ethiopia to I$49.70 in Zambia. Considering the differences in lifespans of each plan, the per capita costs per year ranged from I$0.41 in Ethiopia to I$19.53 in Zimbabwe, and I$46.22 in Nigeria.

All plans will each require less than 1% of the country’s GDP annually for full implementation. The Ethiopian plan is the least burdensome with an annual cost representing 0.01% of the GDP, followed by Senegal at 0.06%. On the other hand, the Nigerian plan was the most expensive at an annual cost representing 0.85% of the country’s GDP, followed by the Zimbabwe plan, which requires 0.77% of the country’s GDP annually for full implementation. Based on the current general government domestic expenditure on health, the Ethiopian, Rwandan, and Senegalese plans would require less than 5% of their current healthcare spending to implement NSOAPs. In contrast, the Nigerian plan would require almost double its current healthcare spending.

In terms of budgetary allocations, Ethiopia, which allocates 2.45% of its annual budget to health needs in 2022, requires 0.11% of its total budget annually to implement its plan. Nigeria, with the allocation of 5%, requires 9.74% of its budget annually to implement its plan. With regards to historic (cumulative) budgetary allocation to health, the total costs of the Rwandan and Ethiopian plans represent about 5% of the total budgetary allocation to health over the entire plan lifespans. This stood at 8.67% and 13.06% respectively in Tanzania and Zambia. In Nigeria, the total costs exceed three times the cumulative budgetary allocation to health over the life span of the plan.

Differences in priorities across countries are reflected in the respective allocations to the same surgical systems domains across the different policies. For example, infrastructure, which constituted around 60% of the costs of the Tanzanian and Zambian plans makes up 33% in the Rwanda plan and only 13% in the Zimbabwe plan. The health workforce constituted comparable proportions of 32–35% of the budgets across all the plans, except in Zambia where personnel development contributed 20%. Overall, leadership/governance and financing domains had the least allocations with each receiving less than 0.5% across all the plans.

**Table 1:** Summary of baseline financing situation across ten countries in Sub-Saharan Africa with National Surgical Obstetrics and Anaesthesia Plans

S/*N*	Country	Resource mobilisation	Financial protection	Purchasing
1	Senegal	The State, local authorities, direct contribution of population, partners in development.	No information	No information
2	Zambia	Surgical, obstetric and anaesthesia (SAO) care, specifically, is funded through public (government), private (households and employers), and external (donor partners) sources.	94% chance of impoverishment from caesarean section, 56% chance of catastrophic expense from surgery	No information
3	Rwanda	85% Community-based health insurance coverage in 2011, formal sector schemes and private insurance account for 6% of the population	50.3% of individuals without health insurance, 6.6% of those covered with any kind of health insurance and on average almost 11% of all Rwandans experience catastrophic expenditure on surgical care, 10% protected against catastrophic expenditure	No information
4	Tanzania	-	85.5% of the population experience impoverishing expenditure on surgical care whilst 65.8% of the population experience catastrophic expenditure	No information
5	Nigeria	Out-of-pocket expenditure − 73%, public spending on healthcare − 12%, health insurance by the National Health Insurance Scheme (NHIS) < 3%, rest as donor funding	65% of the population experience impoverishing expenditure on surgical care and 64% of the population experience catastrophic expenditure	No information
6	Madagascar	Most of the health care spending is from external donors or out-of-pocket costs to patients	13.7% of the population protected against impoverishing expenditure and 4.9% of the population protected against catastrophic expenditure on surgical care	No information
7	Ethiopia	Implementation of health care financing reform (such as fee retention, private wing, service fee revision, etc.), establishment of health insurance, Millennium Development Goals Pool Fund, improving revolving drug fund capital, encouraging multi-sectorial collaborative efforts like National Nutrition Program (NNP), water and sanitation, non-communicable diseases, Ministry of Education	No information	No information
8	Zimbabwe	Comprised of domestic and external resources, resource mobilization through both local and global actors, those covered by the private medical insurance schemes face the challenge of inefficient reimbursement mechanisms as they are usually asked to make co-payments for them to access SAO care	9% of the population experience catastrophic expenditure from surgical care	No information
9	Namibia	The public sector is predominantly funded by the Government of Namibia and serves most of the population. Public health services are utilised by 85% of the population, while the private health sector provides services for a mere 15% of the population. The private sector is funded by private medical aid funds since there is no national health insurance scheme. Despite this dual system, the government reimburses the private sector for services provided to state patients where the service is not available in the public sector. It is important to note that while healthcare in Namibia is theoretically “free of charge” (in that, it is funded by the government) it is expected that the indirect and direct non-medical costs of seeking SOA care are high for individuals.	No information	No information
10	Ghana	Health financing in Ghana is through the Government of Ghana allocation, health insurance from both private and the NHIS, out-of-pocket expenditure and partner support. Health insurance coverage for the population is 68.8%, with the NHIS coverage being 55.4%.	The NHIS and the private health insurance have contributed to reduction of out-of-pocket expenditure on health. However, a large proportion of insured households still experience catastrophic health expenditure. The proportion of the population living below the poverty line dropped by more than half from 52.7% in 1991 to 24.2% in 2013. A quarter (23.4%) of the Ghanaian population earn wages below the national poverty line.	No information

**Table 2:** Costs of National Surgical Obstetrics and Anaesthesia Plans across ten countries in Sub-Saharan Africa

	ZAMBIA	TANZANIA	RWANDA	NIGERIA	ZIMBABWE	SENEGAL	ETHIOPIA	MADAGASCAR	NAMIBIA	GHANA
Year of NSOAP publication	2016	2018	2018	2019	2022	2013	2021	2019	2023	2024
Life span (in years)	5	7	6	5	5	5	5	5	5	5
Updated cost of NSOAP in 2022 (local currency)	6.25Bn	1.2Trn	78.1Bn	7.98Trn	515Bn	51.9Bn	4.3Bn	939Bn	1.03Bn	5.7Bn
Updated cost of NSOAP in I$ in 2022	994.8 M	1.4Bn	227 M	50.5Bn	1.59Bn	221 M	251 M	796 M	155 M	2.2Bn
NSOAP cost/year (in I$)	199 M	200.9 M	37.8 M	10.1Bn	319 M	44.3 M	50 M	159 M	31 M	442 M
NSOAP cost/capita (in I$) in 2022	49.69	21.47	16.45	231.11	97.64	12.78	2.03	26.88	60.42	66.6
NSOAP cost/year/capita	9.94	3.07	2.74	46.22	19.53	2.56	0.41	5.38	12.08	13.32
NSOAP cost/year/capita as a % of GDP/capita in 2022	0.26	0.10	0.10	0.85	0.77	0.06	0.01	0.30	0.11	0.18
Population size, World Bank 2022	20 M	65.5 M	13.8 M	218.5 M	16.3 M	17.3 M	123.4 M	29.6 M	2.6 M	33.1 M
GDP, I$, World Bank 2022	77.96Bn	196.6Bn	38.5Bn	1.28Trn	41.3Bn	72.9Bn	347Bn	52.5Bn	28.8Bn	239Bn
GDP/capita, I$, World Bank 2022	3,894.31	3,096.88	2,792.42	5,432	2,530.65	4,208.96	2,811.58	1,774.07	11,205.71	7,209.04
Domestic general government health expenditure/capita, I$, World Bank 2020	82	43.49	63.83	25.97	26.10	61.07	23.36	21.77		117.21
Domestic general government health expenditure in 2022, I$	1.64Bn	2.85Bn	879 M	5.68Bn	426 M	1.06Bn	2.88Bn	645 M	1.07Bn	3.9Bn
Budgetary allocation to health as a % of total annual budget in 2022	8.03	5.18	7.84	5.00	12.69	-	2.45	-	11.86	8.00
NSOAP cost/year as a % of annual government budget	0.72	0.41	0.28	9.74	11.10	-	0.11	-	0.29	0.83
NSOAP cost/year/capita as a % of domestic general government health expenditure/capita	12.12	7.05	4.30	177.98	74.82	4.19	1.74	24.70	2.89	11.36
Cumulative budgetary allocation to health over 5/6/7 years preceding the plan in local currency (depending on year of launch and the number of years for NSOAP implementation)	47.85Bn	13.86Trn	1.76Trn	2.56Trn	535Bn	-	82Bn	-	37.85Bn	34.65Bn
Total NSOAP cost as a % of cumulative budgetary allocation to health over 5/6/7 years	13.06	8.67	4.43	311.91	96.27	-	5.27	-	2.73	16.38

Abbreviations: GDP, Gross domestic product; I$, International dollars; NSOAP, National surgical, obstetrics and anaesthesia plan

## Discussion

NSOAPs have emerged as a strong signal of national aspiration and political commitment to scale up geographically and financially accessible quality surgery for all citizens. There is now growing momentum across SSA through initiatives such as the Pan African Surgical Healthcare Forum [21]. Delegates participating in the 2023 forum from over 40 countries unanimously agreed that “all African countries should complete their plans/policies at the soonest” while emphasising the need for countries to urgently implement schemes for health insurance that cover all surgical conditions [21]. This aspiration also aligns with high-level agreements, including the strengthening of surgical care as part of World Health Assembly resolution 76.2 (integration of emergency, critical and operative care) passed in 2023. Yet, concerns remain about the progress towards operationalising NSOAPs across all countries [14]. To contribute to our understanding of the potential barriers to the implementation of the plans, we critically analysed the financing and cost content of the expired and existing plans that have been produced in Sub-Saharan Africa. We observed significant differences in structure, scope, and content between the earlier generation policies from Senegal and Ethiopia, which lacked explicit financing-specific objectives and strategies, and later generation plans of Zambia, Tanzania, Nigeria, Rwanda, Madagascar, Zimbabwe, Namibia and Ghana, which featured clearly defined building-blocks-specific strategies (per LCGS recommendation).

Overall, there is a dearth of robust evidence on the implementation of the existing and expired surgical plans in SSA, but the available evidence suggests poor or little implementation with no evidence of tangible improvements in the surgical systems directly attributable to the plans. In Tanzania for instance, an electronic survey to all 26 Regional Health Management Teams (RHMTs) in the mainland to record their insights and understanding of the NSOAP and the state of surgical care showed that only 4% of the RHMTs participated in the NSOAP development process, and 58% were unaware of the policy, and there were no dedicated NSOAP coordinators at the regional level as stipulated in the plan [22]. In Ethiopia, although initial reports indicated strong political commitment and substantial financial allocation to the implementation of SaLTS I [23], a later qualitative assessment of the barriers and facilitators of the development and implementation of the NSOAP showed that the momentum was lost midway due, among other factors, to a change of the Minister of Health of the country [6]. Not surprisingly, Osebo et al. (2022) demonstrated that Ethiopia remains significantly behind in the provision of high quality surgical services measured using the six LCGS surgical situation indicators, several years after the expiration of SALTS I, although their study did not directly assess the implementation of SALTS I [24]. No robust impact evaluation has been conducted and published on any national surgical plan, and it is thought that this is largely due to the fact that no impact evaluation can be conducted where there has been no implementation. Post-term progress reviews of Zambian and Nigerian plans are unpublished but there is no evidence their implementation progressed beyond workshops and trainings [25]. Mid-term progress review of the Rwanda plan found significant improvements in surgical infrastructure across the country but the progress seems to be a collateral benefit of COVID-related investments in general healthcare rather than a result of the NSOAP; the governance structure described in the plan was not set up [26]. Some experts have called for suspension of further efforts in proliferation of national surgical plans till there is a clear evidence of the impact of these plans [27], but the balance of the momentum remains strongly in favour of development of surgical plans [21].

Economics forms the basis of national policies and planning, but economist input remains poor in the surgical planning processes, which are currently predominantly surgeon-centric. Extra-health ministerial stakeholder engagement remains deficient, as critical national institutions often in charge of funding health domains, such as the national Ministry of Finance, are left out. Poor access to surgery is a public health challenge and therefore should be approached as such – leveraging a diverse set of expertise in the planning process. Lessons from the control of infectious diseases efforts suggest that limiting the response to only infectious disease physicians would not have yielded the results achieved with a multi-disciplinary approach [28]. The case of HIV/AIDS response for instance - particularly in SSA - benefited greatly from economic insights in the forms of cost, cost-effectiveness and investment case models that helped justify and attract large-scale funding from international sources like the President’s Emergency Plan for AIDS Relief (PEPFAR) and the Global Fund [29], while helping to shape national policies and secure and sustain political commitment across ministries, including finance and planning [30]. It is crucial to maintain an inter-sectoral engagement and inclusion of non-health political actors (especially legislators who are responsible for appropriation) as well as intensifying public consultations while developing the plans [31].

We identified several international partners involved in supporting the development of the NSOAPs across SSA, with the Harvard PGSSC emerging as the most prominent. The technical assistance provided by these partners has been crucial, not only in offering strategic direction for global surgery, but also in raising awareness, shaping consciousness, and providing a consistent template for surgical systems strengthening across SSA, and such support will remain important moving forward. However, it is increasingly vital that local actors take greater ownership and leadership in the development, adaptation, and implementation of NSOAPs. Regional initiatives such as those led by the Southern African Development Community (SADC) [32], AfroSurg [33], West African College of Surgeons (WACS) [34] and PASHeF [21] represent promising models of locally led collaboration and peer support, fostering contextually grounded solutions and strengthening regional capacity. Locally driven engagement not only enhances the ownership, relevance and sustainability of interventions but also increases the likelihood of successful implementation [35].

We showed that the focus of the financing strategies varied across the NSOAPs, but the Nigerian and Tanzanian plans provided the most elaborate strategies for surgical financing. Most of the plans emphasised resource mobilisation and to a lesser extent financial protection mechanisms. Apart from the NSOAPs put forward by Nigeria and Tanzania, which captured purchasing mechanisms at least implicitly, all other plans failed to focus on purchasing aspects. This lack of attention on the purchasing aspects of global surgery financing in research and policy making has previously been reported as a shortcoming [17, 36]. More attention must be given to the questions of which surgical health services should be bought from whom at which level of the healthcare system, how to pay for these services, and at what rate they should be paid, to ensure that investments in surgical care are value driven [37]. Priority setting exercises through evidence-based deliberative processes could enable determining best buys at various levels across a wide range of interventions [38].

The total costs of NSOAP implementation appear modest when compared with the country’s GDP. However, a comparison with health expenditure raises concerns about the financial viability of the plans. Most countries in SSA still allocate less than the 15 per cent stipulated by the Abuja Declaration to health in their annual budgets [13] (e.g., the 5.75% allocation by Nigeria in 2023 was noted as unprecedented). Earlier analyses suggested that just a modest fiscal space expansion of health expenditure by governments coupled with international development assistance could ensure the full implementation of these plans [16]. However, due to reductions in traditional international development assistance from many key high-income countries, LMICs may be forced to rely more on raising funding through domestic resources.

There has been significant attention to innovative financing as a key strategy for funding of surgical plans in SSA [7]. The Nigerian plan explicitly captured innovative financing options such as Global System of Mobile Communication (GSM) taxes and sin taxes, but an attempt by the Federal Government of Nigeria to implement a nationwide GSM tax faced strong public resistance leading to the suspension of the policy [39]. Moreover, a recent political economy analysis of the opportunities to leverage revenues from existing taxes on sugary drinks revealed a lack of pro-surgery political momentum, suggesting that merely launching a surgical policy is not sufficient [40].

Developing a surgical plan is an important starting point to grow political commitment and outline ambitious targets that can guide strategic investments; however, the implementation of the plan remains the most critical element. There is now a need to start re-imagining the approach to national surgical planning given that existing plans have generally struggled to move beyond the launching ceremony. We contend that the high costs of the plans could contribute at least in part to the low political prioritisation of the NSOAPs in the national resource allocation schemes and hinder integration into national health budgets. A potential solution could involve unbundling and marketing the plans as smaller, less costly implementation packages, aligned with local priorities and health systems capacities. This would enable governments to adopt a phased or modular implementation strategy where smaller allocations of funding are invested in specific bundles in order of priority. For instance, starting with high-impact, politically feasible components such as surgical workforce training or improving access to essential surgical supplies at district hospitals [41]. Specific bundles could also be infused into the budgets of existing more politically favoured vertical programs (e.g., non-communicable diseases, maternal and child health, or primary health care) to leverage existing funding streams. While governments could more easily integrate prioritized components into their annual operational plans and budgets, development partners could take on specific packages aligned with their strategic interests. The modest success observed in Nigeria, where NSOAP activities were translated into partner-specific entry points and a non-governmental organization took up financial responsibility of activities related to paediatric surgery suggests this strategy may improve uptake and implementation [14, 25].

Our study has limitations. First, the policy content analysis was limited to the information provided in the NSOAP documents. It is likely that this misses insights on the experience, challenges, successes, and obstacles encountered by the policy actors and implementers. Further research involving interactions with the different stakeholder on the ground are needed to complement understanding on the experiences not accessible in the policy documents. Second, the scope of our study was restricted to the financing domain of the surgical system and the costing of the NSOAP. By excluding aspects of other surgical system building blocks, we may have inadvertently missed information on costs of the plans which may arise from the interaction across different domains that may be important to foster a comprehensive understanding of the strategies and hence opportunities for improvement. Lastly, the use of Google Translate to translate non-English plans to English Language involves the risk of missing salient points or distortion of the true import of some expressions in the original language. Future studies will benefit from the support of human collaborators who are proficient in both the original publication language and the English language.

## Conclusion

We show that resource mobilisation and financial protection strategies were covered in varying degrees across all ten NSOAPs in SSA, but the purchasing function was notably neglected in all plans. Involvement of (health) economists and the ministry of finance was generally low. The costs of the plans seem to constitute significant obstacles to the implementation of the plans. Future plans should be developed through multi-disciplinary expertise and inter-sectoral engagement with a focus extending beyond resource mobilisation to encompass financial protection and purchasing to ensure sustainable investments in surgical care. Unbundling the plans into smaller contextually ranked packages could render the plans more financially navigable for governments and funders.

## Supplementary Information

Below is the link to the electronic supplementary material.

Supplementary Material 1

Supplementary Material 2

## Data Availability

All data generated or analysed during this study are included in this published article.

## Abbreviations

CBHI: Community-Based Health Insurance
DCP: Disease Control Priorities
GDP: Gross Domestic Product
GSM: Global System of Mobile Communication
LCGS: Lancet Commission on Global Surgery
LMICs: Low- and Middle-Income Countries
NHA: National Health Account
NHIS: National Health Insurance Scheme
NSOAP: National Surgical, Obstetric and Anaesthesia Plan
NSOANP: National Surgical, Obstetric, Anaesthesia and Nursing Plan
PASHef: Pan-African Surgical Healthcare Forum
SOA: Surgery, Obstetrics and Anaesthesia
SSA: Sub-Saharan Africa
UHC: Universal Health Coverage
