# Supplementary material for: National surgical, obstetrics, and anaesthesia plans in Sub-Saharan Africa: a comparative economic content analysis
Source: BMC Health Serv Res. 2026 Jun 29;26:1278. doi: 10.1186/s12913-026-14981-6 (PMC13584366; doi:10.1186/s12913-026-14981-6)
Supplement: Supplementary file 1 [file 12913_2026_14981_MOESM1_ESM.docx]

**Supplementary file 1: Cardno policy document analysis domains and guiding questions**

| S/N | Domain | Guiding questions |
| --- | --- | --- |
| 1 | Document production and location | Why was the document produced?  Where was the document produced and when?  Where was it located?  Was it easy or difficult to access |
| 2 | Authorship and audience | Who wrote the document?  What is their position and do they have a bias?  Who was it written for? |
| 3 | Policy context | What is the purpose of the policy (for the organisation or the state)?  Are drivers or forces behind the policy evident?  What values underpin and guide the policy and are these linked to local or national strategic and quality issues?  Are there multiple values that might create tensions? |
| 4 | Policy text | How is the policy structured and how does the text provide evidence of its construction or development?  What are the key elements of the policy and are they associated with local or national legal or regulatory requirements?  Are there related procedures specified in the text that provide guidance for practice? |
| 5 | Policy consequences | What is the intended overall impact of the policy?  How is policy implementation intended to be monitored?  How and when is the policy to be reviewed?  How does the text draw attention to important aspects of practice related to the policy? |
