# Supplementary material for: National surgical, obstetrics, and anaesthesia plans in Sub-Saharan Africa: a comparative economic content analysis
Source: BMC Health Serv Res. 2026 Jun 29;26:1278. doi: 10.1186/s12913-026-14981-6 (PMC13584366; doi:10.1186/s12913-026-14981-6)
Supplement: Supplementary file 2 [file 12913_2026_14981_MOESM2_ESM.docx]

**Supplemental file 2: Financing goals, strategies, targets, and monitoring and evaluation metrics**

| **Country** | **Overarching goals/objectives** | **Specific action steps/recommendations/strategies** | **Targets/milestones** | **Monitoring and evaluation (metrics tied to specific goals)** |
| --- | --- | --- | --- | --- |
| Senegal | No information | Improve Private-Public Partnership (PPP) to mobilise extra resources to scale up supply of financially accessible surgical services | No information | 1. The number of contracts/agreements shared with private entities 2. The share of the private partners in the funding of the strategic plan |
| Zambia | Improve funding levels and establish budget lines and systems for resource tracking for surgical, obstetric, and anaesthesia services in the country to achieve Universal Health Coverage. | 1. Establish a tracking mechanism for funding of surgical, obstetric, and anaesthesia services:    a. Establish electronic accounting systems for all levels that are linked to MoH headquarters    b. conduct three-yearly surveys to establish the effectiveness of financing mechanisms supporting surgical, obstetric, and anaesthesia services    c. include patient and cashier interviews along with system costs to determine the true cost of surgical, obstetric, and anaesthesia services. 2. Establish budget lines for surgery, obstetrics, and anaesthesia for district hospitals:    a. Create recurrent departmental charges (RDCs) for district hospitals similar to secondary and tertiary hospitals    b. create budget lines for surgery, obstetrics, and anaesthesia at level 1 hospitals as it is currently being done for level 2 and 3 hospitals. 3. Improve funding for surgical, obstetric, and anaesthesia services:    a. Increase the allocation to level 2 and 3 hospital budget lines for surgery, obstetrics, and anaesthesia activities. | Protection against out-of-pocket expenditure for surgery, obstetrics, and anaesthesia should be reduced to zero by 2021 | 1. Financing strategy for SAO developed and implemented by 2021 2. Number of SAO expenditure reports – Tracking mechanisms established and in use in 80% of facilities by 2021 |
| Rwanda | Improve financial protection for patients receiving surgery and increase the allocation of  funds for surgical care | 1. To increase the hospital budget allocated to surgical, obstetric and anaesthesia care 2. To ensure inclusion of all Bellwether procedures among procedures to be covered by Community Based Health Insurance (CBHI) 3. To review of surgical costing index every two years to maintain appropriate costs of services in each region 4. To track funding and expenditures for the NSOAP implementation and review annually through the Health Resource Tracking Tool (HRTT) 5. To intensify resource mobilization to ensure the implementation and sustainability of the NSOAP | 0% of the population protected against impoverishing and catastrophic expenditure | 1. Number of District Hospitals with all bellwether procedures covered by CBHI 2. MOH to review the surgical costing index each year, to maintain appropriate costs of services in regions 3. Conduct a survey every 3 years to establish the effectiveness of health insurance coverage for SAO care 4. Annually review the budget allocated to SAO care |
| Tanzania | 1. Ensure adequate funding to support NSOAP activities 2. Decrease catastrophic and impoverishing expenditure resulting from surgery | 1. Assess the current funding that SOA receives:    a. undertake a thorough mapping of current SAO resources including national, regional, and local government and partners. 2. Advocate for SAO needs throughout Tanzania at the national level:    a. Engage Parliamentarian Champions e.g.(Safe motherhood group, Benjamin Mkapa foundation, social services committee)    b. engage influential people and celebrities    c. showcase the complementary role of NSOAP in RMNCH and align with RMNCH priorities    d. showcase the role of SAO care as critical to the treatment of NCDs and align with priorities in these areas 3. Advocate for SAO needs throughout Tanzania from the council level:    a. ensure the joint Annual health sector review is sensitized to SAO needs    b. NSOAP representative/Coordinator to advocate for SOA needs at the TWG annual health sector review    c. speak through joint Annual health sector review and policy review meetings    d. advocate for surgery inclusion in CCHPs 4. Advocate for new funding sources for SAO care:    a. advocate for the establishment of a Trauma fund from motor insurance companies    b. advocate for part of road fund for trauma care,    c. Lobby for NHIF to allow funds to be used for the purchase of surgical consumables 5. Match costs and reimbursement for surgical care for sustainable scale-up:    a. map costs of select SAO procedures across each zone/region    b. advocate for realistic reimbursements of SAO care from health insurance companies    c. establish out-of-pocket/co-payment where reimbursement of full cost is achieved based on pricing index    d. establish bundled payments for surgical services so patients can estimate full expense prior to receiving elective care 6. Develop partnerships with private providers and private enterprises to sponsor SAO programs:    a. identify potential external and pprivate funders    b. NSOAP committee and MoF to approach external and private funders to advocate for funding of programs within the NSOAP 7. Iimprove insurance coverage for surgical conditions:    a. work with health insurance companies to ensure the inclusion of core surgical procedures with appropriate cost in the UHC package    b. advocate health insurance companies around the cost-effectiveness of surgical care and its important role in attaining UHC to make a case for coverage 8. Work with the social services department to sensitize communities about the cost of surgical conditions:    a. develop a strategy for community sensitization around the cost of SAO care and the availability of insurance coverage | 1. Increasing enrolment in social health insurance schemes from 19% in 2015 to 50% by 2020. 2. Increase the share of total government expenditure allocated to health from 9.1% in 2015 to 10.0% by 2020 3. 30% of the annual NSOAP budget from domestic funds committed at a national level to SAO 4. 30% of the annual NSOAP budget from funds committed to SAO included in CCHPs 5. All procedure costs being met by reimbursement and co-payment 6. 35% of the total NSOAP budget from funds committed to NSOAP activities from external partners 7. 70% of the population is protected from catastrophic financial risk due to surgery | 1. Resource mapping completed by 2018 2. Domestic funds committed at a national level to SAO 3. Funds committed to SAO included in Comprehensive Council Health Plans (CCHPs) 4. Alternative funding sources secured (amount in TZS) 5. Proportion of full procedure costs being met by reimbursement and co-payment 6. Funds committed to NSOAP activities from external partners |
| Nigeria | Achieve financial risk protection for 50% of the population by 2023 | 1. Increase financial risk protection:    a. Strengthening the legal framework establishing the National Health Insurance Scheme (NHIS) to make health insurance mandatory    b. working assiduously towards establishing mandatory health schemes in all the 36 states of the federation and the FCT    c. strengthening the capacity of States with mandatory health schemes to utilize earmarked funds released for the provision of basic health care under the National Health Act    d. designing a model that allows for pensioners under the contributory pension scheme (holders of Retirement Saving Accounts) to be included and covered under a vulnerable programme of the NHIS with zero co-payment    e. deepen the National Health Insurance Fund by mandating the deduction at source of 1.75% of the consolidated salary of all employees of the Federal Government covered by the NHIS 2. Reduce out-of-pocket expenditure:    a. Expand health insurance coverage in terms of benefit package that covers surgical service inclusive of the treatment of congenital anomalies requiring advanced surgical procedures 3. Innovative funding for healthcare:    a. Introducing a Global System for Mobile (GSM) communication tax of one kobo per second on all mobile telephone calls    b. Implementing the proposed sin tax on tobacco and alcohol with the resultant proceeds earmarked for investing in the healthcare system    c. Making concerted efforts in line with the established federal government policy on Public/ Private Partnership for Health, by shopping for supply-side financing of expensive health equipment and infrastructure at single-digit interest rates    d. Integrating parallel activities of donor bodies and corporate social responsibility (CSR) of businesses into a singular effort to boost demand-side financing for health 4. Increase efficiency gains in healthcare:    a. Cost-cutting collaborations by government to reduce duplication of effort in the healthcare system    b. Reduction of activities with limited impact on health targets such as ‘free’ health programmes that do not have policy and legal backing, and channel funds usually allocated to such into health insurance pools for a more sustainable venture    c. Entrenching fiscal discipline in the system to ensure that budgetary allocation and release of funds are matched with periodic monitoring of the utilization of the same    d. Performance-based budgeting for public healthcare facilities, where allocations and some allowances are tied to performance to reduce the frequency of industrial actions and increase productivity    e. Employ Strategic Purchasing as an allocation tool as is obtainable under health insurance systems where services rather than line items or commodities are budgeted for to address the challenge of skewed budgeting 5. Make healthcare an investment priority:    a. The Nigerian healthcare system requires a paradigm shift in policy-making that would make funding of healthcare an investment with quantifiable returns in terms of human capital preservation and revenue generation to make Nigeria a medical tourism hub as obtainable in India and Israel | 1. 25% out-of-pocket spending rate on surgical care 2. 40% health insurance coverage rate 3. Increase budgetary allocation to health to 10% 4. Achieve an 80% implementation rate for capital expenditure on health. | 1. Impoverishing expenditure rate 2. Catastrophic expenditure rate 3. Percentage of out-of-pocket expenditure for healthcare 4. Percentage of health budget mobilized from the private sector 5. Cost-effectiveness of interventions 6. Percentage budgetary allocation to health |
| Madagascar | No information | 1. Optimize the management of usual financial resources:    a. Advocacy with competent institutions (Parliament, Government, etc.) to increase the budget of the Ministry of Public Health to 16% at the national level for the benefit of the "hospital care programme"    b. Bring financial managers of establishments up to standard hospitals in terms of programming and budget execution    c. Honor in the finance law of the consolidated budgets of each hospital establishment validated by a competent financial structure of the Ministry    d. Solicit the participation of CTDs in the management of medical care especially for the poor    e. Encourage the implementation of an innovative system to increase revenue and improve the quality of care    f. Intensify and systematize monitoring, auditing, and financial supervision at the level of public hospitals 2. Develop the partnership:    a. Advocacy with Technical and Financial Partners (local, traditional and other) on financing the development of hospitals    b. Support public hospitals in the search for partners    c. Institutionalize the “Contract of Objectives and Means” approach | 1. 16% increase in the budget of the Ministry of Public Health by 2023 2. 100% of hospitals to develop and apply an innovative system for increasing revenue and improving the quality of care | 1. Percentage increase in the budget of the Ministry of Public Health 2. Percentage of financial managers trained in programming and budget execution 3. Percentage of the consolidated budgets of each hospital establishment validated by a competent financial structure of the Ministry 4. Percentage of CTDs having an account in their budget for the provision of medical care, particularly for the poor 5. Percentage of Hospital centres that have developed and applied an innovative system for increasing revenue and improving the quality of care 6. Percentage of Hospital centres audited during the year 7. Percentage of institutions with financiers 8. Percentage of Hospital centres benefiting from support in the search for partners 9. Regulatory text on the institutionalization of the "Contract of Objectives and Means” published |
| Ethiopia | No information | 1. Provide and allocate financial support at all levels of the health system  2. Promote the PPP model to ensure equitable and affordable surgical care (flagship initiative) | No information | National Health Accounts (NHA) to be used as a health financial burden assessment tool that will be deployed in monitoring progress in household protection from financial toxicities due to surgery |
| Zimbabwe | Improve funding levels and establish budget lines and systems for resource tracking for SOA services to achieve Universal Health Coverage | 1. Establish the cost of SOA services to determine the funding levels required for SOA care services:    a. Conduct a costing exercise of all SOA services 2. Improve funding for SOA services and strengthen resource mobilization from insurance providers:    a. Define SOA Packages at all levels for the Essential Health Benefit    b. Develop a SOA Investment Case that links to the National Health IC and is based on the Essential SOA Package    c. Increase the allocation of funding on budget lines for SOA activities at district-level hospital    d. Review the user fees exemption policies to ensure all with the ability to pay are made to pay e.g. clients covered by medical insurance including those under 5, over 65, maternity patients and victims of RTA    e. Implement a real-time validation of the membership remittances system    f. Ensure that the hospitals are reimbursed by the central government for SOA services rendered to exempted groups 3. Implement measures to reduce wastage of resources:    a. Establish a standardized risk and loss control system at all hospitals 4. Efficient use of available resources:    a. Capacitate Procurement Management Unit to be able to procure goods and services in a cost-effective manner    b. Review and expedite implementation of the National Health Insurance (NHI) Roadmap. | No information | No information |
| Namibia | Protect patients from impoverishing indirect and direct non-medical costs of SOA and critical care. | To evaluate and respond to patient impoverishment due to service requirements:   a. Establish a programme for the evaluation of patient costs and out-of-pocket- payments (OOPs) b. Community engagement programme focused on cost reductions | No information | Proportion of facilities reporting patient incurred costs for SOA care |
| Ghana | Provide sustainable financing for SOTA care through diversified funding mechanisms and deployment  of cost-effective care models | 1. Enhance affordability of safe, effective and high-quality SOTA care and protect against catastrophic and impoverishing health expenditure:    a. Include all Bellwether procedures in the NHIS    b. Register and maintain members on health insurance    c. Review surgical costing index 2. Improve resource mobilization and budget allocation for SOTA    a. Develop resource mobilization plan for NSOAP    b. Provide subvention for implementation and sustainability of NSOAP    c. Increase budget allocated to SOTA care | 100% throughout the 5 years  90% by Year 5 | Proportion of Bellwether  procedures fully covered by  the NHIS  1.Proportion of households  protected against catastrophic  expenditure from direct out-of pocket payments for surgical  and anaesthesia care  2. Proportion of households  protected against impoverishment from direct  out-of-pocket payments for  surgical and anaesthesia  care  Reviewed surgical costing  index available  Resource mobilization plan for NSOAP available  Amount of funds allocated for  implementation of NSOAP  received  Surgical expenditure as a  proportion of total national  healthcare budget  Surgical expenditure as a  proportion of GDP |

**Abbreviations**: CBHI, Community-based health insurance; CCHP, Comprehensive council health plan; CSR, Corporate social responsibility; FCT, Federal capital territory; GDP, Gross domestic product; GSM, Global System for Mobile; HRTT, Health resource tracking tool; MOH, Ministry of health; NCD, Non-communicable disease; NHA, National health account; NHIF, National health insurance fund; NHIS, National health insurance scheme; NSOAP, National surgical, obstetric and anaesthesia plan; OOP, Out-of-pocket; PPP, Public-private partnership; RDC, Recurrent departmental charges; RMNCH, Reproductive maternal, newborn and child health; SAO, Surgery, anaesthesia and obstetric; SOA, Surgery, obstetric and anaesthesia; SOTA, Surgery, obstetric, trauma and anaesthesia; TWG, Technical working group; TZS, Tanzanian Shillings; UHC, Universal health coverage.
